# Pulmonary Metastasectomy After Liver Transplantation: Indications, Timing, and Surgical Decision-Making Across Primary Tumor Histologies

**DOI:** 10.3390/diagnostics16152397

**Published:** 2026-07-30

**Authors:** Vasiliki Androutsopoulou, Dimitrios E. Magouliotis, Vanesa Brecher, Noah Sicouri, Dimitrios Zacharoulis, Fabrizio Minervini, Ugo Cioffi, Marco Scarci

**Affiliations:** 1 Department of Cardiothoracic Surgery, Faculty of Medicine, University of Thessaly, Biopolis, 41110 Larissa, Greece; androutsopoulouvasiliki@uth.gr; 2 Department of Cardiac Surgery Research, Lankenau Institute for Medical Research, Main Line Health, Wynnewood, PA 19096, USA; magouliotisd@mlhs.org (D.E.M.); vb4075@pcom.edu (V.B.); nps67@pitt.edu (N.S.); 3 Department of Surgery, Faculty of Medicine, University of Thessaly, Biopolis, 41110 Larissa, Greece; zacharoulis@uth.gr; 4 Department of Thoracic Surgery, Luzern Kanton Hospital, 6000 Luzern, Switzerland; fabriziominervini@hotmail.com; 5 Department of Surgery, University of Milan, 20157 Milan, Italy; ugo.cioffi@guest.unimi.it; 6 Department of Cardiothoracic Surgery, Hammersmith Hospital, Imperial College Healthcare, National Health Service (NHS) Trust, London W2 1NY, UK

**Keywords:** liver transplantation, pulmonary metastasectomy, hepatocellular carcinoma, colorectal liver metastases, lung resection, transplant oncology, patient selection, immunosuppression

## Abstract

Liver transplantation (LT) has evolved from a treatment for end-stage benign liver disease into a therapeutic strategy for selected oncological indications, including hepatocellular carcinoma (HCC), unresectable colorectal liver metastases (CRLM), hepatoblastoma, neuroendocrine tumor hepatic metastases, and hepatic epithelioid hemangioendothelioma. As the indications for LT expand and post-transplant survival improves, pulmonary recurrence has emerged as the predominant pattern of extrahepatic relapse across histologies. Yet no standardized surgical guidelines exist for managing lung metastases in the post-transplant patient. This narrative review synthesizes the available evidence on pulmonary metastasectomy following LT, addressing the incidence and tumor-specific patterns of pulmonary recurrence, the influence of immunosuppression on metastatic biology, surgical indications and contraindications, approach and timing considerations, and patient selection across primary histologies. We propose a practical decision-making framework integrating primary tumor biology, disease-free interval, lesion characteristics, immunosuppression regimen, and systemic therapy response. With the recent regulatory recognition of CRLM as a standard transplant indication and the ongoing SECA-III randomized trial, the downstream surgical management of pulmonary recurrence in LT recipients requires urgent evidence-based codification.

## 1. Introduction

Liver transplantation (LT) represents one of the most profound transformations in modern surgical oncology. Originally confined to benign end-stage hepatic disease, the field underwent a paradigm shift with the introduction of the Milan criteria in 1996, which established HCC as a curative indication for LT within defined morphological boundaries [1]. Over the subsequent three decades, the conceptual scope of transplant oncology has widened considerably. Unresectable CRLM, neuroendocrine hepatic metastases, hepatic epithelioid hemangioendothelioma (HEHE), and hepatoblastoma are now recognized transplant indications at experienced centers, and the regulatory framework is evolving to reflect this reality [2].

The Norwegian SECA-I trial enrolled 21 patients with unresectable CRLM and demonstrated a 5-year overall survival (OS) of 60%, fundamentally challenging the assumption that metastatic colorectal cancer precluded transplantation [3,4]. Subsequent iterations refined patient selection through the Oslo score, and SECA-II achieved a 5-year OS of 83% under more stringent criteria [5]. The TRANSMET trial confirmed the superiority of LT over systemic chemotherapy in a randomized design, reporting a median OS exceeding 50 months in the transplant arm [6]. Most recently, in July 2025, updated Organ Procurement and Transplantation Network guidance allowed selected patients with unresectable CRLM to be considered for MELD exception review through the National Liver Review Board [2,7], an important development for transplant oncology.

A consistent and clinically significant observation across all these trials and registries is that pulmonary recurrence constitutes the dominant pattern of extrahepatic relapse following LT for oncological indications. In SECA-I and SECA-II, approximately 70% of recurrences manifested in the lung, and a substantial proportion of these patients were amenable to further surgical resection with favorable downstream survival [3,5]. This pattern is not limited to CRLM: post-transplant pulmonary metastases are well-described in HCC, neuroendocrine tumors, and pediatric hepatic tumors, each with distinct biology and surgical implications [8,9].

Despite this, pulmonary metastasectomy in the post-transplant patient remains without standardized surgical guidelines. The unique physiological context of the transplant recipient, including chronic immunosuppression, altered hepatic and renal function, drug interactions, and potential immunotherapy contraindications, adds complexity that generic pulmonary oncology protocols do not address. Moreover, the accelerating expansion of LT indications means that the volume of post-transplant patients developing lung recurrence will grow substantially in coming years. This narrative review synthesizes the available evidence on pulmonary metastasectomy following LT across primary tumor histologies, and proposes a structured decision-making framework to guide surgical selection in this growing and underserved patient population.

Search strategy: For this narrative review we searched PubMed/MEDLINE, Scopus, and Web of Science from January 2000 to June 2026, combining the terms “liver transplantation”, “pulmonary metastasectomy”, “lung metastasis”, “hepatocellular carcinoma”, “colorectal liver metastases”, “neuroendocrine tumor”, “hepatoblastoma”, “hepatic epithelioid hemangioendothelioma”, “immunosuppression”, and “mTOR inhibitor”. Original clinical studies, randomized trials, and registry analyses reporting outcomes of pulmonary resection or local ablative therapy after LT were prioritized; narrative reviews, consensus statements, and mechanistic studies were included where primary data were sparse, and reference lists of retrieved articles were hand-searched for additional reports. Given the rarity of the condition, no formal quality weighting or meta-analytic pooling was undertaken, and evidence across primary tumor histologies was synthesized qualitatively. This approach is descriptive rather than systematic, and the resulting synthesis and proposed framework should be interpreted as expert opinion informed by the available, predominantly retrospective, literature.

## 2. Pulmonary Recurrence as the Dominant Pattern After Liver Transplantation

### 2.1. Incidence and Biological Basis

The predilection for pulmonary recurrence following LT for oncological indications reflects both anatomical and biological factors. The lungs represent the first capillary bed encountered by tumor cells entering the systemic circulation from a hepatic primary, establishing a favorable environment for metastatic seeding. For HCC, pulmonary recurrence occurs in approximately 20–40% of patients who develop post-transplant relapse, constituting the most common site of extrahepatic spread [10]. For CRLM, the lung parenchyma emerges as the dominant site precisely because LT eliminates hepatic disease while systemic micrometastatic deposits may persist [5]. Immunosuppression plays a critical and underappreciated role in post-transplant metastatic biology. Calcineurin inhibitors carry pro-oncogenic properties mediated through upregulation of transforming growth factor-beta, stimulation of vascular endothelial growth factor, and impaired cancer immunosurveillance, creating a permissive environment for accelerated metastatic growth at pulmonary sites [11,12].

### 2.2. The mTOR Inhibitor Shift

A pivotal advance in post-transplant oncological immunosuppression has been the substitution of calcineurin inhibitors with mammalian target of rapamycin (mTOR) inhibitors, specifically sirolimus and everolimus [11]. The SiLVER trial, a randomized controlled study in HCC transplant recipients, demonstrated that conversion to sirolimus-based immunosuppression was associated with improved recurrence-free survival, particularly in patients within Milan criteria [13]. mTOR inhibitors exert antiproliferative effects through inhibition of the PI3K/AKT/mTOR pathway, which is dysregulated in many solid tumors, and confer direct antiangiogenic activity [12]. In the post-transplant setting, early conversion to an mTOR inhibitor-based regimen may be considered on an individualized basis for patients with oncological transplant indications, particularly when pulmonary surveillance detects early recurrence. This recommendation should nonetheless be individualized rather than applied reflexively: the decision to convert to, or add, an mTOR inhibitor at the time of pulmonary recurrence must weigh graft function, rejection risk, renal function, wound-healing and perioperative infection considerations, and primary tumor type, and should be made jointly with the transplant team rather than as a default surgical prescription. The Italian Liver Transplant Working Group 2024 consensus recommends mTOR inhibitor incorporation as part of oncological immunosuppression protocols, and a systematic review published in 2025 confirmed that mTOR-based regimens are associated with reduced recurrence across multiple transplant oncology indications [12]. This immunosuppression context is directly relevant to the surgical management of pulmonary metastases: the decision to resect should be coordinated with a formal review and potential modification of the immunosuppression regimen.

**Figure 1:**
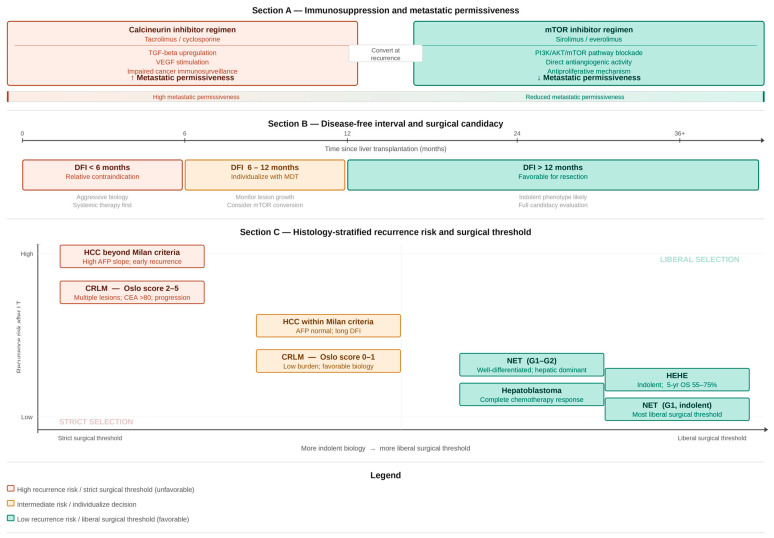
Biological determinants of pulmonary recurrence and surgical eligibility after liver transplantation. The figure is organized across three sections. Section A contrasts the two principal immunosuppression paradigms: calcineurin inhibitor-based regimens (tacrolimus, cyclosporine), which upregulate TGF-beta and VEGF and impair cancer immunosurveillance, thereby increasing metastatic permissiveness, and mTOR inhibitor-based regimens (sirolimus, everolimus), which block the PI3K/AKT/mTOR pathway, exert direct antiangiogenic and antiproliferative activity, and reduce metastatic permissiveness. Conversion at the time of pulmonary recurrence may be considered on an individualized basis, weighing graft function, rejection risk, and tumor type. Section B maps surgical candidacy across the post-transplant timeline according to disease-free interval (DFI): DFI below 6 months represents a relative contraindication warranting systemic therapy evaluation first; DFI of 6 to 12 months requires individualized multidisciplinary decision-making with attention to lesion growth rate and consideration of mTOR conversion; DFI exceeding 12 months is favorable and supports full candidacy evaluation for resection. Section C positions primary tumor histologies on a two-dimensional plane according to recurrence risk after LT (vertical axis) and surgical threshold stringency (horizontal axis). Histologies characterized by aggressive biology (HCC beyond Milan criteria, CRLM with Oslo score 2–5) occupy the high-risk, strict-threshold quadrant. Intermediate-risk histologies (HCC within Milan criteria, CRLM with Oslo score 0–1) occupy the central zone. Indolent histologies (NET G1–G2, hepatoblastoma, HEHE, and indolent G1 NET) occupy the low-risk, liberal-threshold quadrant, supporting more permissive surgical selection. AFP, alpha-fetoprotein; CEA, carcinoembryonic antigen; CNI, calcineurin inhibitor; CRLM, colorectal liver metastases; DFI, disease-free interval; HCC, hepatocellular carcinoma; HEHE, hepatic epithelioid hemangioendothelioma; LT, liver transplantation; MDT, multidisciplinary team; mTOR, mammalian target of rapamycin; NET, neuroendocrine tumor; OS, overall survival; PI3K/AKT, phosphoinositide 3-kinase/protein kinase B; SBRT, stereotactic body radiotherapy; TGF-beta, transforming growth factor beta; VEGF, vascular endothelial growth factor. Created in BioRender. Magouliotis, D. (2026) https://BioRender.com/iswkpbe (accessed on 4 May 2026).

The three determinants discussed in this section, namely immunosuppression class, disease-free interval, and primary tumor histology, interact to define the overall metastatic risk landscape and surgical eligibility of each post-transplant patient. Figure 1 illustrates this conceptual framework, mapping immunosuppression-modified metastatic permissiveness, DFI-stratified candidacy zones, and histology-stratified recurrence risk across the post-transplant continuum, providing a visual synthesis of the biological rationale underpinning patient selection.

### 2.3. Diagnostic Evaluation of Suspected Pulmonary Recurrence

Because surgical decision-making is only as sound as the diagnostic pathway that precedes it, the recognition and characterization of pulmonary recurrence in the LT recipient deserve explicit attention. Post-transplant pulmonary nodules are typically first detected on surveillance contrast-enhanced computed tomography (CT) of the chest, which we suggest at 3- to 6-month intervals for oncological LT indications, with the interval individualized to recurrence risk. No transplant-specific surveillance schedule has been prospectively validated, and this interval reflects pragmatic expert opinion rather than guideline-based evidence. A newly detected nodule in an immunosuppressed transplant recipient cannot be assumed to be metastatic: the differential diagnosis is broad and includes bacterial, fungal (e.g., *Aspergillus, Cryptococcus*), and mycobacterial infection, organizing pneumonia, and post-transplant lymphoproliferative disorder, all of which may mimic metastatic disease radiologically [14]. Features favoring metastasis include a well-circumscribed, peripheral, non-cavitary nodule that enlarges on serial imaging in a patient with a rising tumor marker, whereas cavitation, a surrounding ground-glass halo, rapid multifocal appearance with systemic symptoms, or interval response to antimicrobial therapy should prompt an infectious or inflammatory work-up before oncological intervention.

18F-FDG PET/CT may contribute to staging, but its ability to distinguish malignancy from infection or inflammation is limited in the immunosuppressed host, where infective and inflammatory lesions are frequently FDG-avid, so it does not reliably exclude an infectious cause and active infection must be considered independently. Tumor markers should be interpreted in a histology-specific manner: a rising alpha-fetoprotein in HCC [15] or carcinoembryonic antigen in CRLM increases the pre-test probability of true recurrence, while chromogranin A and 68Ga-DOTATATE PET are relevant for neuroendocrine tumors. Circulating tumor DNA is an emerging adjunct that may flag recurrence before it is radiologically overt [16] (Section 8.1). Histological confirmation by CT-guided core biopsy is warranted when the diagnosis is uncertain, when infection cannot be excluded, when the result would change management (for example, before committing an immunosuppressed patient to thoracic surgery), or when tissue is required for molecular profiling; biopsy is generally deferred when imaging, marker trajectory, and multidisciplinary consensus already establish a high probability of resectable oligometastatic recurrence, since a negative percutaneous sample does not exclude malignancy [17] and resection is both diagnostic and therapeutic. This diagnostic sequence, from surveillance detection through differential diagnosis, functional imaging, and selective biopsy to multidisciplinary confirmation, is intended to precede the surgical selection framework presented in Section 7.

## 3. HCC Recurrence: Pulmonary Metastasectomy After Liver Transplantation for Hepatocellular Carcinoma

### 3.1. Epidemiology and Timing of Pulmonary Recurrence

HCC recurrence after LT occurs in 15–25% of patients transplanted within Milan criteria, and in higher proportions when expanded criteria are applied [10]. Pulmonary metastases represent the most common site of extrahepatic recurrence, often manifesting as solitary or oligometastatic nodules on computed tomography surveillance. The pattern of recurrence carries meaningful prognostic implications: early recurrence (within 12 months of LT) typically reflects aggressive tumor biology with microvascular invasion or satellite lesions not captured in pre-transplant staging, while late pulmonary recurrence may indicate a more indolent phenotype amenable to curative-intent surgery [10].

Pre-transplant alpha-fetoprotein (AFP) trajectory has emerged as one of the most powerful predictors of post-transplant recurrence risk. The Duvoux model, which incorporates AFP slope alongside tumor size and number, identifies patients at highest recurrence risk and can inform the intensity of post-transplant surveillance protocols [15]. Patients transplanted with AFP greater than 1000 ng/mL or a rising AFP slope despite locoregional therapy carry substantially higher recurrence risk and may warrant more frequent pulmonary imaging. With respect to the nature of pulmonary recurrence, HCC lung metastases after LT most often present as peripheral, well-defined nodules that are single or few in number at the earliest detectable stage, typically becoming apparent between 12 and 36 months post-transplant, although earlier recurrence within the first year is described and portends worse biology. Multiplicity, central location, and bilateral distribution generally emerge later and correlate with a more aggressive phenotype and greater symptom burden, with corresponding impact on quality of life once dyspnea, cough, or hemoptysis supervene. Beyond AFP, candidate predictors of pulmonary-specific recurrence include the AFP slope and the AFP-L3 and des-gamma-carboxyprothrombin (PIVKA-II) fractions, microvascular invasion and poor differentiation on explant pathology, tumor burden beyond Milan criteria, and, increasingly, circulating tumor DNA dynamics; none is yet validated as a lung-specific biomarker, and prospective study is needed.

### 3.2. Evidence for Surgical Resection

The landmark Italian multicenter experience reported outcomes for pulmonary metastasectomy in HCC patients following LT at three transplant centers [8] (Table 1). In this retrospective cohort, patients who underwent pulmonary resection for isolated HCC pulmonary metastases achieved a median OS exceeding 30 months after metastasectomy, compared with significantly shorter survival in non-surgically managed patients. R0 resection was the strongest predictor of favorable outcome, and patients with solitary lesions and a disease-free interval exceeding 12 months demonstrated the best results [8]. A Korean single-center experience evaluated pulmonary metastasectomy specifically in LT recipients with HCC recurrence [18]. Among patients who underwent complete resection, 5-year survival rates after metastasectomy approached 40%, substantially exceeding historical benchmarks for systemic therapy alone in this population. The authors identified oligometastatic disease (three or fewer lesions), unilateral distribution, and AFP normalization following resection as the most favorable prognostic features [18].

Systemic therapy context matters considerably. Sorafenib demonstrated no survival benefit as adjuvant therapy following curative resection or ablation of hepatocellular carcinoma in the STORM trial [19]. No randomized data directly compare systemic therapy with pulmonary metastasectomy in this setting, and any preference for resection in resectable oligometastatic recurrence rests on indirect evidence and expert opinion rather than on direct comparison. The integration of more modern systemic agents, including atezolizumab-bevacizumab combinations, with surgical management of pulmonary recurrence remains under investigation, though immunotherapy must be approached with particular caution in transplant recipients given the risk of graft rejection.

## 4. The CRLM Paradigm: Pulmonary Recurrence After Liver Transplantation for Colorectal Liver Metastases

### 4.1. The SECA-TRANSMET Framework

Colorectal cancer is the third most common malignancy worldwide, and approximately 40–50% of patients will develop hepatic metastases during the course of their disease [20]. While resectable CRLM are potentially curable with hepatectomy, unresectable disease carries a dismal prognosis with systemic therapy alone. SECA-I demonstrated that LT for unresectable CRLM achieved a 5-year OS of 60%, far exceeding chemotherapy benchmarks [3,4]. SECA-II achieved a 5-year OS of 83% under more stringent Oslo score-based selection [5]. The TRANSMET trial confirmed the superiority of LT over chemotherapy in a randomized design [6]. Most recently, in July 2025, updated Organ Procurement and Transplantation Network guidance allowed selected patients with unresectable CRLM to be considered for MELD exception review through the National Liver Review Board [2,7].

A defining feature of CRLM transplant oncology is the high rate of pulmonary recurrence combined with preserved survival after surgical management of that recurrence. In SECA-I and SECA-II, approximately 70% of recurrences were pulmonary, and the majority of these patients could be offered lung surgery, with post-recurrence OS of 73% at 4 years [5]. This paradox, high disease recurrence but favorable OS, reflects the oligometastatic and biologically indolent phenotype of recurrences in well-selected CRLM transplant recipients [21]. As with HCC, the characterization of pulmonary recurrence in CRLM after LT is clinically informative. Lung metastases in this setting are frequently oligometastatic and peripheral, often detected on surveillance imaging within the first two to three years, and the SECA experience indicates that many such patients remain candidates for resection or ablation with preserved survival. Predictors of pulmonary recurrence and of post-recurrence outcome include the Oslo score, pre-transplant CEA level, primary tumor sidedness and RAS/BRAF mutational status, metabolic tumor burden on pre-transplant FDG-PET/CT, and post-transplant ctDNA kinetics, the last of which may identify impending pulmonary relapse before it becomes radiologically or symptomatically manifest.

### 4.2. Patient Selection: The Oslo Score and Predictive Tools

The Oslo score integrates five factors: maximum tumor diameter, number of liver metastases, carcinoembryonic antigen (CEA) level, performance status, and progression on pre-transplant chemotherapy [21] (Table 2). Patients with low Oslo scores (0–1) demonstrated 5-year OS of 75% after LT, and critically, even those who developed pulmonary recurrence frequently achieved durable disease control with subsequent resection [21]. Grut and colleagues demonstrated that pre-transplant FDG-PET/CT uptake within the liver metastases predicts both early pulmonary recurrence and response to post-recurrence lung surgery, establishing a functional imaging biomarker relevant to surgical planning [22]. Bjornsson and colleagues further showed that higher standardized uptake values in the liver lesions prior to transplantation correlated inversely with survival after pulmonary metastasectomy, suggesting that the biological aggressiveness encoded in the primary hepatic deposit projects forward onto the prognosis of subsequent pulmonary lesions [23].

### 4.3. Re-Resection and the SECA-III Design

The SECA-III trial is a randomized comparison of LT versus multidisciplinary standard-of-care in unresectable CRLM patients [24]. The trial design is registered as ClinicalTrials.gov NCT03494946 [24], and the stepwise conceptual approach underpinning it has been described previously [25]. A distinctive and surgically relevant design feature is the inclusion of patients with resectable pulmonary metastases at the time of transplant listing, permitting combined LT and pulmonary metastasectomy or staged sequential procedures. This design acknowledges the clinical reality observed in SECA-I, namely that long-term survivors frequently required lung surgery and that iterative resection was safe and beneficial [26]. The feasibility of iterative hepatic and pulmonary resections in the non-transplant CRC population has been confirmed in a 2025 multicenter analysis, which demonstrated comparable complication profiles across successive resections with maintained survival benefit [27]. While direct extrapolation to the transplant population requires caution given the immunosuppression context, the principle that re-resection of pulmonary oligometastases is safe and effective in appropriately selected patients appears durable across surgical contexts.

## 5. Other Histologies

### 5.1. Neuroendocrine Tumors

LT for unresectable hepatic metastases from neuroendocrine tumors (NET) is an established indication, typically restricted to well-differentiated G1-G2 tumors with hepatic-dominant disease and absent extrahepatic spread at listing [9]. Pulmonary metastases following LT for NET reflect the characteristically indolent biology of these tumors and are often detected as late, asymptomatic findings on surveillance imaging. Mazzaferro and colleagues demonstrated long-term benefit from LT in this population, with 5-year OS exceeding 70% in selected patients [9]. When pulmonary recurrence occurs post-LT, the favorable tumor biology may favor surgical resection in patients who are otherwise fit, consistent with the broader evidence for pulmonary metastasectomy in NET patients. Direct evidence for pulmonary metastasectomy specifically after LT in NET is essentially absent; the transplant literature cited here supports the transplant indication and long-term survival rather than post-transplant pulmonary resection, and any recommendation is extrapolated from non-transplant NET metastasectomy series and should be regarded as indirect.

### 5.2. Hepatoblastoma

Hepatoblastoma is the most common primary hepatic malignancy in children under 5 years, and LT is indicated for unresectable disease without extrahepatic metastases at the time of transplantation [28]. Pulmonary metastases represent the dominant site of distant relapse, and limited evidence, largely from small pediatric series, supports surgical resection of pulmonary lesions prior to LT listing as a prerequisite for transplant candidacy, as well as resection of recurrent pulmonary lesions after LT [28]. The integration of chemotherapy, lung surgery, and LT in a multimodal sequential strategy has produced long-term survivors in patients with otherwise catastrophic prognosis. Evidence for pulmonary metastasectomy after LT in hepatoblastoma is limited to small pediatric series and multimodal case reports; the cited literature describes hepatoblastoma treatment and transplant outcomes rather than post-transplant pulmonary resection specifically, so these recommendations rest on indirect data.

### 5.3. Hepatic Epithelioid Hemangioendothelioma

HEHE is a rare vascular tumor with indolent behavior and 5-year survival of 55–75% following either resection or LT [29]. Pulmonary involvement may be present at the time of LT listing in some patients and does not necessarily preclude transplantation given the indolent trajectory of this malignancy. When pulmonary disease develops or progresses post-LT, surgical resection may be considered on an individualized basis in the context of tumor biology and pace of progression, recognizing that the natural history of HEHE differs fundamentally from that of carcinomas. Direct evidence for pulmonary metastasectomy after LT in HEHE is essentially absent and limited to isolated case reports; the cited literature addresses HEHE outcomes and transplantation rather than post-transplant pulmonary resection, so any surgical recommendation reflects indirect data and individualized judgment.

## 6. Surgical Considerations: Approach, Timing, and Technical Aspects

### 6.1. Operative Approach

Video-assisted thoracoscopic surgery (VATS) has become the preferred approach for pulmonary metastasectomy in the general oncological population, offering comparable oncological outcomes to thoracotomy with reduced morbidity, shorter hospital stay, and faster recovery [30]. In the post-transplant patient, the arguments for a minimally invasive approach are amplified: reduced surgical stress, lower immunosuppression perturbation, diminished risk of infectious complications in an immunocompromised host, and faster return to baseline immunosuppression dosing. For bilateral synchronous or sequential pulmonary metastases, staged VATS procedures or simultaneous bilateral VATS can be considered in experienced centers [30].

The evidence base for VATS metastasectomy in the transplant-specific population remains limited to retrospective series, and no randomized data directly compare VATS to open resection in this context [31]. However, the trajectory of surgical oncology practice strongly favors VATS as the default approach for resectable oligometastatic pulmonary disease. Conversion to thoracotomy is warranted for central lesions, those requiring sleeve resection, or cases with extensive pleural adhesions from prior cardiothoracic procedures.

### 6.2. Timing and Disease-Free Interval

The disease-free interval (DFI), defined as the time from LT to detection of pulmonary recurrence, is one of the most consistent prognostic factors across histologies [32]. A DFI of 12 months or greater has been associated with significantly improved survival after pulmonary metastasectomy in both HCC-transplant and CRLM-transplant populations [8,32]. Short DFI (less than 6 months) suggests aggressive tumor biology, rapid immunosuppression-facilitated proliferation, or occult systemic disease at the time of LT, and should prompt reassessment of resection candidacy. Early pulmonary recurrence warrants systemic therapy evaluation and multidisciplinary tumor board review before committing to resection. Late recurrence (beyond 12 months) with oligometastatic disease in a fit patient generally supports resection, particularly when R0 is achievable.

### 6.3. Perioperative Management in the Transplant Patient

Perioperative management of LT recipients undergoing thoracic surgery requires coordination between the thoracic surgeon, hepatologist or transplant team, and anesthesiologist [33]. Key considerations include adjustment of calcineurin inhibitor levels in the perioperative period, drug interactions between immunosuppressants and anesthetic agents, risk of infectious complications mandating strict prophylactic antimicrobial coverage, and the need to avoid nephrotoxic agents in patients with pre-existing calcineurin inhibitor-related renal impairment [33]. Immunosuppression modification at the time of pulmonary metastasectomy represents a clinical opportunity: conversion or addition of mTOR inhibitor therapy may be considered on an individualized basis after discussion with the transplant team, and where adopted, the timing of any adjustment should be individualized rather than fixed to a defined preoperative window; the aim is to harness the antiproliferative properties of sirolimus or everolimus alongside their immunosuppressive mechanisms [13].

## 7. Patient Selection Framework

### 7.1. Proposed Decision Algorithm

**Figure 2:**
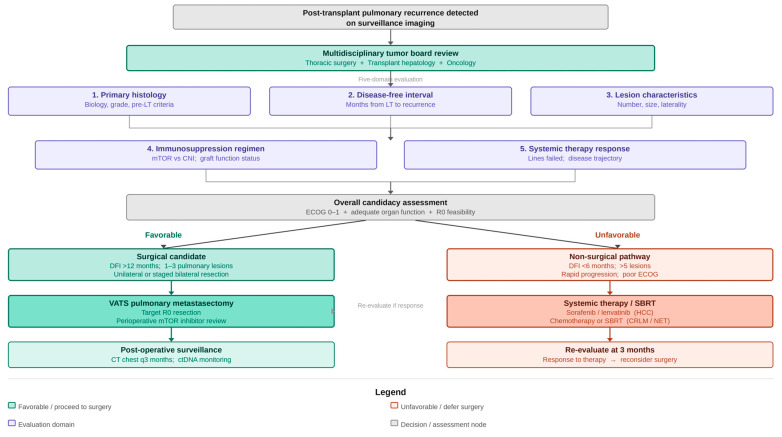
Patient selection algorithm for pulmonary metastasectomy after liver transplantation. Following detection of post-transplant pulmonary recurrence on surveillance imaging, candidates undergo multidisciplinary tumor board review involving thoracic surgery, transplant hepatology, and oncology. Evaluation proceeds across five domains in parallel: primary tumor histology and biology; disease-free interval (DFI); pulmonary lesion characteristics; immunosuppression regimen; and systemic therapy response. Findings are integrated into an overall candidacy assessment incorporating performance status, organ function, and technical resectability. Favorable candidates are directed to the surgical arm, which proceeds through VATS pulmonary metastasectomy targeting R0 resection with perioperative mTOR inhibitor review, followed by post-operative surveillance with CT chest every three months and ctDNA monitoring. Unfavorable candidates enter the non-surgical arm and receive systemic therapy or stereotactic body radiotherapy, with structured re-evaluation at three months and feedback to surgical candidacy if a clinical response is achieved. The numeric thresholds depicted (for example, disease-free interval, lesion number, and organ-function cut-offs, together with the three-month surveillance and reassessment intervals) are pragmatic, expert-opinion values that have not been externally validated and are intended to structure multidisciplinary discussion rather than to serve as strict or independent decision rules. CNI, calcineurin inhibitor; ctDNA, circulating tumor DNA; DFI, disease-free interval; ECOG, Eastern Cooperative Oncology Group; HCC, hepatocellular carcinoma; LT, liver transplantation; MDT, multidisciplinary team; mTOR, mammalian target of rapamycin; NET, neuroendocrine tumor; R0, complete resection; SBRT, stereotactic body radiotherapy; VATS, video-assisted thoracoscopic surgery. Created in BioRender. Magouliotis, D. (2026) https://BioRender.com/p295xs1 (accessed on 4 May 2026).

Based on the synthesized evidence, we propose a structured decision framework for pulmonary metastasectomy in LT recipients organized around five domains: (1) primary tumor histology and biology; (2) disease-free interval; (3) lesion characteristics; (4) immunosuppression regimen; and (5) systemic therapy response prior to surgical consideration (Table 3). This framework is intended as a guide for multidisciplinary tumor board decision-making rather than a prescriptive algorithm. We emphasize that the five-domain framework and the accompanying figures and tables represent expert opinion synthesized from predominantly retrospective, single-center, and indirect evidence, not an externally validated, evidence-based clinical pathway; the specific thresholds proposed (for example, disease-free interval and lesion-number cut-offs) are pragmatic constructs intended to structure discussion rather than validated decision rules. Equally important, the primary tumor histologies considered here, including HCC, CRLM, neuroendocrine tumors, hepatoblastoma, and hepatic epithelioid hemangioendothelioma, differ substantially in biology, recurrence kinetics, systemic treatment, and prognosis, and a single unified framework necessarily abstracts away these differences. The domains should therefore be applied through a histology-specific lens, informed by the tumor-specific sections above, and should not be read as implying equivalent evidence or identical thresholds across these distinct diseases. Figure 2 presents the complete decision pathway, from detection of post-transplant pulmonary recurrence through the five evaluation domains, branching into the surgical arm, which proceeds to VATS metastasectomy and post-operative surveillance, and the non-surgical arm, which directs patients toward systemic therapy or SBRT with structured re-evaluation at three months.

Favorable candidates for pulmonary metastasectomy share the following profile across histologies: DFI exceeding 12 months; three or fewer pulmonary lesions; unilateral distribution or bilateral disease amenable to staged resection; radiographically stable or slowly progressive lesions; performance status ECOG 0–1; adequate hepatic and renal function; and absence of extrathoracic extrahepatic disease [32,34]. Tumor histology modifies these thresholds: HEHE and well-differentiated NETs may warrant more liberal surgical thresholds given their indolent biology, while poorly differentiated tumors or those with early recurrence post-LT should be approached conservatively.

### 7.2. Systemic Therapy Integration

Conventional cytotoxic chemotherapy is generally applicable with standard dosing in transplant recipients, though hepatotoxicity monitoring is essential and calcineurin inhibitor levels must be monitored more frequently given pharmacokinetic interactions. Targeted agents, particularly bevacizumab, should be withheld for at least four weeks before and after surgery given the risk of anastomotic complications and impaired wound healing [35]. Checkpoint inhibitor immunotherapy carries a significant risk of graft rejection through enhanced T-cell-mediated alloreactivity [36]. For HCC recurrence, sorafenib or lenvatinib remain the pragmatic systemic options in the transplant population, as the atezolizumab-bevacizumab combination that has become first-line in non-transplant patients [37] cannot be reflexively applied in this context.

### 7.3. Contraindications to Resection

Absolute contraindications to pulmonary metastasectomy in LT recipients include extrathoracic extrahepatic disease that is not controlled or resectable, uncontrolled graft rejection or active graft dysfunction, active systemic infection, and performance status precluding safe thoracic surgery. Relative contraindications warranting multidisciplinary review include DFI below 6 months, more than five pulmonary lesions, bilateral disease with compromised expected residual lung function, and rapid progression through systemic therapy prior to surgical referral [32,34].

## 8. Future Directions and Conclusions

### 8.1. Liquid Biopsy and Surveillance

Circulating tumor DNA (ctDNA) and liquid biopsy technologies are emerging as powerful surveillance tools in the post-transplant oncological context [16]. In the CRLM transplant population, ctDNA positivity post-LT may precede radiological evidence of recurrence by months, enabling earlier detection of pulmonary micrometastases and potentially informing the decision to initiate or modify systemic therapy before overt surgical-stage disease develops [16]. The integration of ctDNA-based surveillance into post-LT oncological monitoring protocols represents a high-priority research agenda.

### 8.2. Stereotactic Body Radiotherapy as an Alternative

Stereotactic body radiotherapy (SBRT) has established a growing role in the management of pulmonary oligometastases in patients who are not surgical candidates [38]. In the post-transplant population, where surgical risk may be elevated by immunosuppression-related comorbidities, SBRT offers a non-invasive alternative for patients with resectable but surgically high-risk pulmonary lesions. The absence of head-to-head comparisons between SBRT and VATS metastasectomy in the LT population is a critical evidence gap, and pragmatic registry studies are needed to define the optimal modality for specific lesion and patient profiles. Several LT-specific practicalities merit emphasis. First, the safety of thoracic SBRT in chronically immunosuppressed recipients is incompletely defined: radiation pneumonitis is the principal dose-limiting toxicity, and immunosuppression together with prior pulmonary infection or fibrosis may modify that risk, so careful attention to lung dose-volume constraints and exclusion of active infection is essential; in the randomized SABR-COMET trial, stereotactic radiotherapy improved survival but was associated with treatment-related death in a small proportion of patients, underscoring that it is not without risk [39]. Second, although no LT-specific dose-fractionation schedule has been established, commonly employed peripheral lung oligometastasis regimens deliver a biologically effective dose above 100 Gy (for example, 48 to 54 Gy in three to five fractions), with more fractionated schedules for central lesions to respect proximal bronchial and vascular tolerance; these should be adapted to the immunosuppressed context and agreed upon within the multidisciplinary team. Third, published SBRT outcomes specific to the post-LT population are essentially absent, and this evidence gap should be acknowledged when counseling patients. Finally, for patients who are resectable but at high surgical risk, percutaneous image-guided thermal ablation, including microwave ablation, radiofrequency ablation, and cryoablation, provides an additional local-control option; multicenter data such as the SOLSTICE cryoablation study demonstrate favorable local control and an acceptable safety profile for pulmonary metastases in the general oncological population, although, as with SBRT, transplant-specific evidence and comparative data against metastasectomy are lacking [40].

### 8.3. Prospective Registries and Trial Design

The evidence base for pulmonary metastasectomy in LT recipients is currently limited to retrospective single-center and multicenter series, with inherent selection bias. The establishment of prospective international registries capturing post-transplant oncological outcomes, including the surgical management of extrahepatic recurrence, is essential to generate the higher-quality evidence needed to refine selection criteria and surgical strategies. SECA-III provides a model for integrating pulmonary recurrence management into transplant trial design and may yield randomized data relevant to this question [24].

### 8.4. Conclusions

Pulmonary metastasectomy after liver transplantation represents an underrecognized and underpracticed therapeutic opportunity in a patient population that is growing as transplant oncology indications expand. Pulmonary metastasectomy may be considered in highly selected LT recipients across several primary tumor histologies, and reported 5-year survival after resection is encouraging; its independent survival benefit, however, remains uncertain given the retrospective, selection-biased nature of the available evidence. This apparent benefit must, however, be interpreted with caution: the supporting data derive almost entirely from retrospective series subject to substantial selection bias, in which patients chosen for metastasectomy tend to have better performance status, lower tumor burden, and more indolent disease biology than those managed non-operatively, and no randomized comparison isolates the effect of surgery itself. Surveillance, systemic therapy, SBRT, and percutaneous ablation remain legitimate alternatives in appropriately selected patients, and the framework proposed here is intended to structure multidisciplinary deliberation rather than to assert the superiority of surgery. Key selection factors include favorable primary tumor biology, DFI exceeding 12 months, oligometastatic unilateral or resectable bilateral disease, and adequate organ function. The unique biological context of chronic immunosuppression, particularly the opportunity to leverage mTOR inhibitor conversion in the perioperative period, adds a dimension absent from general pulmonary oncology practice. As CRLM transplantation enters mainstream practice following recent OPTN regulatory changes, the downstream surgical management of pulmonary recurrence requires urgent prospective study and evidence-based codification. Multidisciplinary tumor board oversight, early involvement of dedicated thoracic surgical expertise, and coordination with the transplant team are prerequisites for optimal outcomes in this complex patient population.

## Figures and Tables

**Table 1:** Summary of published series on pulmonary metastasectomy after liver transplantation for hepatocellular carcinoma recurrence.

Study	n	Surgical Approach	Median OS After Metastasectomy	Key Prognostic Factors
Invernizzi et al. [8] (Italian multicenter, 2020)	21	Open thoracotomy/VATS	>30 months	R0 resection; DFI > 12 months; solitary lesion; AFP normalization
Jeong et al. [18] (Korean single-center, 2021)	18	VATS preferred	5-year OS ~40%	Lesion count ≤ 3; unilateral disease; AFP normalization post-resection; DFI > 12 months

AFP, alpha-fetoprotein; DFI, disease-free interval; OS, overall survival; VATS, video-assisted thoracoscopic surgery.

**Table 2:** Oslo score variables and SECA trial program outcomes for liver transplantation in unresectable colorectal liver metastases.

Variable/Trial	Definition/Threshold	Outcome/Score
Oslo Score Components		
Max tumor diameter	≤55 mm vs. >55 mm	0 vs. 1 point
Number of liver metastases	≤2 vs. >2	0 vs. 1 point
CEA level	≤80 ng/mL vs. >80 ng/mL	0 vs. 1 point
Performance status	ECOG 0 vs. ECOG ≥1	0 vs. 1 point
Progression on chemotherapy	No vs. yes	0 vs. 1 point
Score interpretation	Score 0–1: favorable; score 2–5: unfavorable	5-yr OS: 75% (score 0–1) vs. 37% (score 2–5) [21]
SECA Trial Program		
SECA-I [4]	21 patients; unresectable CRLM; no Oslo score selection	5-yr OS 60%; ~70% of recurrences pulmonary; majority resectable
SECA-II [5]	15 patients; Oslo score 0 required; stringent selection	5-yr OS 83%; DFS 35% at 3 yr; post-recurrence OS 73% at 4 yr [5]
TRANSMET [6]	Randomized vs. chemotherapy; French multicenter	Median OS > 50 months (LT) vs. 25 months (chemo)
SECA-III [24]	Randomized; includes patients with resectable lung metastases at LT listing	Ongoing; first RCT integrating pulmonary metastasectomy in LT trial design

CEA, carcinoembryonic antigen; CRLM, colorectal liver metastases; DFS, disease-free survival; ECOG, Eastern Cooperative Oncology Group; LT, liver transplantation; OS, overall survival; RCT, randomized controlled trial.

**Table 3:** Proposed five-domain patient selection framework for pulmonary metastasectomy after liver transplantation.

Domain	Favorable (Supports Resection)	Unfavorable (Reassess/Systemic Therapy First)
1. Primary histology and biology	HCC within Milan criteria at LT; well-differentiated NET (G1–G2); HEHE; hepatoblastoma with complete chemotherapy response; CRLM with low Oslo score	Poorly differentiated HCC; high AFP slope at LT; CRLM with Oslo score ≥ 2; rapidly progressive primary
2. Disease-free interval	DFI > 12 months: strongly favorable	DFI < 6 months: relative contraindication; DFI 6–12 months: individualize with MDT
3. Lesion characteristics	≤3 lesions; unilateral; radiologically stable or slow-growing; R0 achievable; no pleural effusion	>5 lesions; bilateral with compromised residual lung function; rapid radiological progression; pleural or mediastinal involvement
4. Immunosuppression regimen	mTOR inhibitor-based (sirolimus/everolimus) or convertible; stable graft function; no active rejection	Active rejection; unstable immunosuppression; contraindication to mTOR inhibitor; severe calcineurin inhibitor nephrotoxicity limiting perioperative management
5. Systemic therapy response	No prior systemic therapy (surgery-naive); stable disease on systemic therapy; complete/partial radiological response	Progression through ≥ 2 lines of systemic therapy; rapidly progressive despite treatment; ctDNA strongly rising
Performance and organ function	ECOG 0–1; adequate hepatic (Child-Pugh A) and renal function; no active infection	ECOG ≥ 2; hepatic decompensation; significant renal dysfunction; uncontrolled systemic infection

ctDNA, circulating tumor DNA; DFI, disease-free interval; ECOG, Eastern Cooperative Oncology Group; HCC, hepatocellular carcinoma; HEHE, hepatic epithelioid hemangioendothelioma; LT, liver transplantation; MDT, multidisciplinary team; mTOR, mammalian target of rapamycin; NET, neuroendocrine tumor. The favorable and unfavorable descriptors and any numeric thresholds shown are pragmatic, expert-opinion constructs intended to structure multidisciplinary discussion. They have not been externally validated in the post-transplant pulmonary metastasectomy population and should not be applied as strict or independent decision criteria. The three-month intervals shown for postoperative surveillance and for reassessment during systemic or ablative therapy are likewise pragmatic suggestions rather than validated schedules. The evidence base also differs substantially across the histologies represented: direct data for pulmonary metastasectomy after liver transplantation are largely limited to hepatocellular carcinoma and colorectal liver metastases and are essentially absent for neuroendocrine tumors, hepatoblastoma, and hepatic epithelioid hemangioendothelioma.

## Data Availability

No new data were created or analyzed in this study. Data sharing is not applicable to this article.

## Abbreviations

Abbreviation	Full Term
AFP	Alpha-fetoprotein
AKT	Protein kinase B
CEA	Carcinoembryonic antigen
CNI	Calcineurin inhibitor
CRC	Colorectal cancer
CRLM	Colorectal liver metastases
CT	Computed tomography
ctDNA	Circulating tumor DNA
DFI	Disease-free interval
DFS	Disease-free survival
ECOG	Eastern Cooperative Oncology Group
eGFR	Estimated glomerular filtration rate
FDG-PET/CT	Fluorodeoxyglucose positron emission tomography/computed tomography
HCC	Hepatocellular carcinoma
HEHE	Hepatic epithelioid hemangioendothelioma
LT	Liver transplantation
MDT	Multidisciplinary team
mTOR	Mammalian target of rapamycin
NET	Neuroendocrine tumor
OPTN	Organ Procurement and Transplantation Network
OS	Overall survival
PD-1	Programmed cell death protein 1
PD-L1	Programmed death-ligand 1
PI3K	Phosphoinositide 3-kinase
R0	Complete (margin-negative) resection
RCT	Randomized controlled trial
SBRT	Stereotactic body radiotherapy
SECA	Scandinavian Exceptional Cases Allocation (trial series)
SiLVER	Sirolimus in Liver Transplant Recipients With Hepatocellular Carcinoma (trial)
STORM	Sorafenib as Adjuvant Treatment in the Prevention of HCC Recurrence (trial)
SUV	Standardized uptake value
TGF-beta	Transforming growth factor beta
TRANSMET	Liver Transplantation for Colorectal Cancer Liver Metastases (trial)
VATS	Video-assisted thoracoscopic surgery
VEGF	Vascular endothelial growth factor
